# Fabrication of Epitaxial Fe_3_O_4_ Film on a Si(111) Substrate

**DOI:** 10.1038/s41598-017-07104-z

**Published:** 2017-08-01

**Authors:** Nozomi Takahashi, Teodor Huminiuc, Yuta Yamamoto, Takashi Yanase, Toshihiro Shimada, Atsufumi Hirohata, Taro Nagahama

**Affiliations:** 1 Graduate School of Chemical Sciences and Engineering, Hokkaido University, Sapporo, 060-8628 Japan; 2 Department of Physics, University of York, Heslington, York, YO10 5DD England; 3 Graduate School of Engineering, Hokkaido University, Sapporo, 060-8628 Japan; 4 Department of Electronic Engineering, University of York, Heslington, York, YO10 5DD England

## Abstract

The application of magnetic oxides in spintronics has recently attracted much attention. The epitaxial growth of magnetic oxide on Si could be the first step of new functional spintronics devices with semiconductors. However, epitaxial spinel ferrite films are generally grown on oxide substrates, not on semiconductors. To combine oxide spintronics and semiconductor technology, we fabricated Fe_3_O_4_ films through epitaxial growth on a Si(111) substrate by inserting a γ-Al_2_O_3_ buffer layer. Both of γ-Al_2_O_3_ and Fe_3_O_4_ layer grew epitaxially on Si and the films exhibited the magnetic and electronic properties as same as bulk. Furthermore, we also found the buffer layer dependence of crystal structure of Fe_3_O_4_ by X-ray diffraction and high-resolution transmission electron microscope. The Fe_3_O_4_ films on an amorphous-Al_2_O_3_ buffer layer grown at room temperature grew uniaxially in the (111) orientation and had a textured structure in the plane. When Fe_3_O_4_ was deposited on Si(111) directly, the poly-crystal Fe_3_O_4_ films were obtained due to SiO_x_ on Si substrate. The epitaxial Fe_3_O_4_ layer on Si substrates enable us the integration of highly functional spintoronic devices with Si technology.

## Introduction

In the field of spintronics, spin injection and transport phenomena have attracted much attention owing to the possibility of producing novel functional devices^1–3^. In particular, the combination of spintronics and semiconductors is a promising technology for the development of the next stage of spintronic devices, e.g., spin-FET or logic devices^4, 5^. The spin injection technique, in which the spin-polarized currents are injected from ferromagnetic metals into conventional semiconductor materials^2, 3, 6^, has been intensely investigated for the preparation of spintronic devices. As a result, researchers have succeeded in nonlocal detection^7^ or the observation of the Hanle effect^1^, which demonstrates the spin state in the semiconductor; thus, the behavior of the spin current in the semiconductor can be determined^8^. Recently, graphene has also been the subject of spin injection because the spin diffusion length in such light elements is expected to be long owing to small spin–orbit interaction^9, 10^.

The source of the spin current plays an important role in obtaining high-efficiency spin injection. Magnetic oxides are one of the most promising spin source candidates. However, ferromagnetic metals have been used so far because of convenience during fabrication. Magnetic oxides possess unique properties^11–14^; Fe_3_O_4_ or (LaSr)MnO_3_ have a half-metallic state, which provides highly spin polarized current^15^, and NiFe_2_O_4_ or CoFe_2_O_4_ are magnetic insulators, which means that they could work as a spin filter tunnel barrier^16–18^. γ-Fe_2_O_3_ is another candidate as the spin filter barrier. It is the spinel type ferrimagnetic insulator that is obtained by over oxidation of Fe_3_O_4_^19^. Recently, NiCo_2_O_4_ with spinel structure was discovered to exhibit large magnetoresistance effects^20^. Therefore, the combination of magnetic oxides and semiconductors enables us to produce new functional devices. Some research groups fabricated the magnetic oxide on oxide semiconductor, Nb:SrTiO_3_, and investigated the transport characteristics including spin transport of the junctions^19, 21^. However, epitaxial growth of magnetic oxide on Si, which is the most important semiconductor, has not been established because the surface of Si is easily oxidized by the oxygen atmosphere during the evaporation of the magnetic oxides^22^.

In this study, we grew Fe_3_O_4_ epitaxially on a Si(111) substrate by the insertion of an ultrathin γ-Al_2_O_3_ buffer layer. Fe_3_O_4_ is the ferrimagnetic conducting oxide with spinel crystal structure. At 120K, Fe_3_O_4_ shows phase transition called Verwey transition, at which the electric resistivity increases drastically and the crystal symmetry decreases from face-centered cubic to monoclinic^23–25^. Fe_3_O_4_ is expected to be half-metallic theoretically, meaning to have a spin polarization of 100%^15^, and a spin polarization of more than 80% was observed experimentally using a spin-resolved photoemission spectroscopy^26^. An ultrathin γ-Al_2_O_3_ layer was inserted to prevent surface oxidation of Si during the Fe_3_O_4_ growth. γ-Al_2_O_3_ is an aluminum oxide with the same spinel structure as Fe_3_O_4_ and the lattice constant of γ-Al_2_O_3_ is 7.91 Å, which is three halves of that of Si with lattice mismatch of −2.9%^27^. From the viewpoint of the crystal structure, Fe_3_O_4_ and γ-Al_2_O_3_ seems to grow on Si epitaxially.

γ-Al_2_O_3_ (111) epitaxial growth on Si(111) was reported by two research groups recently. Jung *et al*. formed a γ-Al_2_O_3_ (111) layer by annealing an Al layer on protective Si oxide, which was carefully oxidized to be reduced by the Al layer^28^. Merckling *et al*. fabricated γ-Al_2_O_3_ (111) by the deposition of an Al_2_O_3_ source under ultra-high vacuum^29^. In the former method, it is difficult to optimize the oxidation of the Si layer and the thickness of Al film. In contrast, the latter method is simple if an ultra-high vacuum system is accessible.

In this study, the epitaxial γ-Al_2_O_3_ buffer layers were prepared using an ultra-high vacuum system and the Fe_3_O_4_ layer was fabricated by reactive molecular beam epitaxy. We investigated the crystal structure, magnetic and electric properties of the Fe_3_O_4_ layer on Si(111) with an epitaxial γ-Al_2_O_3_ buffer layer, an amorphous-Al_2_O_3_ buffer layer, and without a buffer layer. We succeeded in the fabrication of high quality Fe_3_O_4_ films on Si(111) substrates. The buffer layer had a significant effect on the crystal structure of the Fe_3_O_4_ layers.

## Results and Discussion

### Epitaxial growth

**Figure 1:**
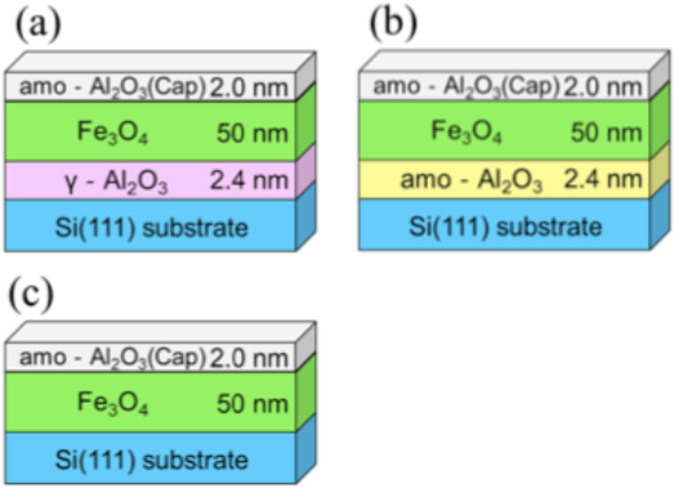
Sample structures: (**a**) the sample with γ-Al_2_O_3_ buffer layer (EPI), (**b**) with amorphous-Al_2_O_3_ buffer layer (AMO), and (**c**) without a buffer layer (W/O).

**Figure 2:**
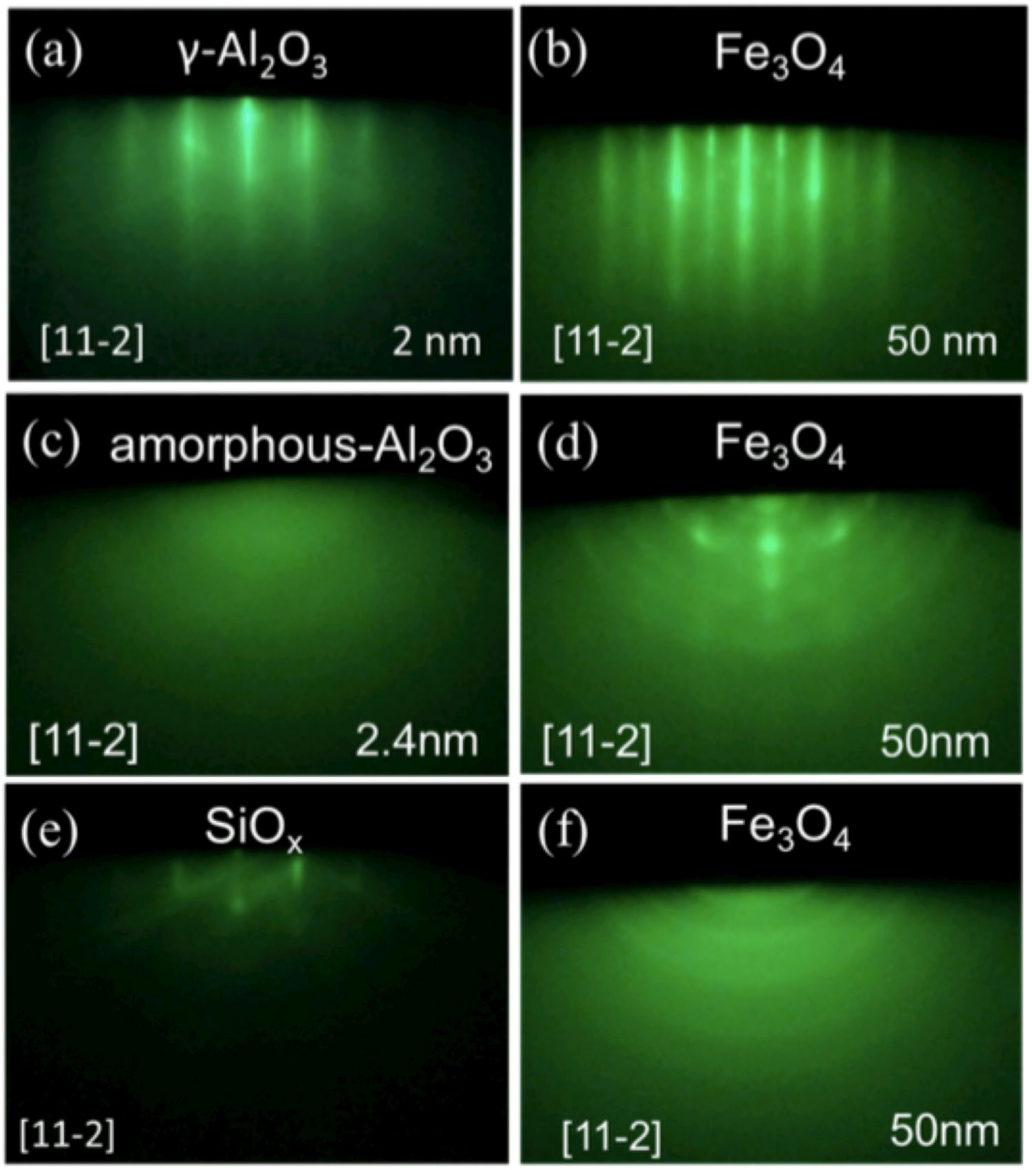
RHEED patterns: (**a**) γ-Al_2_O_3_ and (**b**) Fe_3_O_4_ film on γ-Al_2_O_3_ in EPI, (**c**) amorphous-Al_2_O_3_ and (**d**) Fe_3_O_4_ on amourphous-Al_2_O_3_ in AMO, (**e**) Si surface before depositing Fe_3_O_4_, and (**f**) Fe_3_O_4_ on Si substrate in W/O.

The γ-Al_2_O_3_ and Fe_3_O_4_ layers were grown by molecular beam epitaxy method. The structures of the samples were (a) Si(111)/γ-Al_2_O_3_ 2.4 nm/Fe_3_O_4_ 50 nm/amorphous-Al_2_O_3_ 2.0 nm, (b) Si(111)/amorphous-Al_2_O_3_ 2.4 nm/Fe_3_O_4_ 50 nm/amorphous-Al_2_O_3_ 2.0 nm and (c) Si(111)/Fe_3_O_4_ 50 nm/amorphous-Al_2_O_3_ 2.0 nm, as shown in Fig. 1 (hereafter referred to as (a) EPI, (b) AMO and (c) W/O), respectively. After treatment of the Si substrate, we confirmed that the *in-situ* reflection high energy electron diffraction (RHEED) pattern of the Si substrate had a (7 × 7) streak pattern (Supplementary Fig. S1). This means that the surface of Si was clean and flat. Figure 2(a) and (b) show the RHEED pattern of γ-Al_2_O_3_ and Fe_3_O_4_ in EPI. The direction of the incident electron beam was [11-2]. The RHEED patterns of γ-Al_2_O_3_ and Fe_3_O_4_ were clear streak patterns indicating that γ-Al_2_O_3_ and Fe_3_O_4_ grew epitaxially. Therefore, the γ-Al_2_O_3_ film was considered to play a role of a buffer layer for epitaxial growth of Fe_3_O_4_. The surface roughness of γ-Al_2_O_3_ and Fe_3_O_4_ were estimated to be very small in value by atomic force microscope (AFM) (shown in Supplementary Fig. S2).

Figure 2(c) and (d) show the RHEED pattern of amorphous-Al_2_O_3_ and Fe_3_O_4_ in AMO. The amorphous-Al_2_O_3_ layer was deposited at room temperature. After the deposition of Al_2_O_3_, as shown in Fig. 2(c), the Si (7 × 7) streak pattern turned into a halo pattern, which indicated that the Al_2_O_3_ layer was amorphous. Figure 2(d) shows the RHEED pattern of Fe_3_O_4_ on the amorphous-Al_2_O_3_. A ring and streak pattern was observed, which implied the presence of a polycrystalline surface. Thus, the epitaxial γ-Al_2_O_3_ played a crucial role in the formation of epitaxial Fe_3_O_4_ on the Si substrate.

Figure 2(e) and (f) show the RHEED pattern of the Si substrate and Fe_3_O_4_ in W/O. The surface of the Si substrate exhibited a diffused streak pattern owing to the introduction of oxygen gas, which oxidized the Si surface. In Fig. 2(f), the RHEED pattern of Fe_3_O_4_ on SiO_x_ shows a halo pattern, which indicated that spinel-type Fe_3_O_4_ was not formed.

### X-ray diffraction

**Figure 3:**
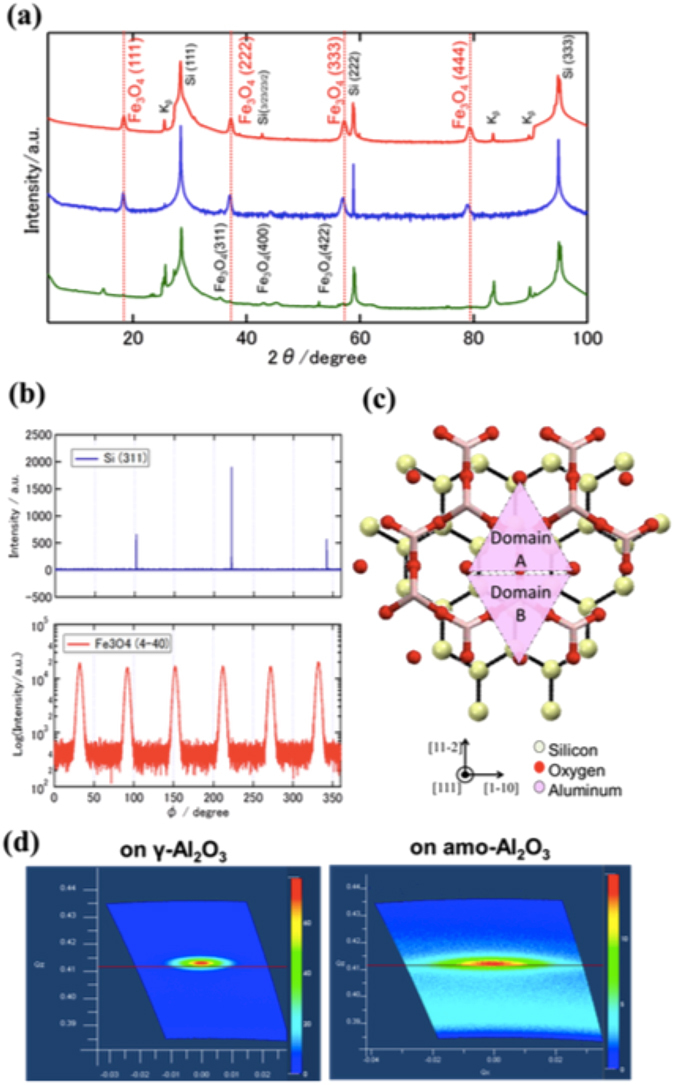
X-Ray diffraction: (**a**) θ-2θ XRD profiles, (**b**) ϕ-scan measurement, (**c**) Epitaxial relationship between Si and γ-Al_2_O_3_, (**d**) X-ray reciprocal space maps of Fe_3_O_4_ on γ-Al_2_O_3_ in EPI and Fe_3_O_4_ on amo-Al_2_O_3_ in AMO.

To confirm the crystallization, the θ–2θ X-ray diffraction (XRD) measurements were carried out on three samples, as shown in Fig. 3(a). The diffraction pattern of Fe_3_O_4_ on an γ-Al_2_O_3_ buffer layer in EPI (red line) exhibited four peaks (18.3°, 37.2°, 57.2°, 79.4°), which were in agreement with the diffraction patterns of Fe_3_O_4_ (111), (222), (333) and (444) planes. This indicated that the Fe_3_O_4_ film was (111)-oriented without other orientations or phases. The lattice constant measured by XRD was estimated to be 8.39 Å. The lattice constant of the in-plane direction was estimated to be 8.23 Å (Supplementary Fig. S3), which is smaller than the bulk lattice parameter. Therefore, the Fe_3_O_4_ was considered to be compressed in-plane.

To investigate the in-plane epitaxial relationship, we conducted ϕ-scan measurements of Si(311) and Fe_3_O_4_ (4-40), as shown in Fig. 3(b). The six peaks of Fe_3_O_4_ (4-40) appeared at 60° intervals, indicating the presence of two 180° rotated domains in the Fe_3_O_4_ layer. The epitaxial relationships were [11-2]Fe_3_O_4_(111) and [-1-12]Fe_3_O_4_(111) parallel to [11-2]Si(111), as exhibited in Fig. 3(c). In addition, the peaks of the Fe_3_O_4_ film were broader than that of the Si substrate. There was a lattice mismatch of 5.7% at γ-Al_2_O_3_/Fe_3_O_4_.

The θ–2θ XRD diffraction pattern of Fe_3_O_4_ in AMO (blue line) exhibited four peaks, which was identical with the diffraction pattern of Fe_3_O_4_ in EPI. Therefore, the Fe_3_O_4_ in AMO was also (111)-oriented. However, the RHEED pattern in Fig. 1(d) implied the presence of a polycrystalline structure. Furthermore, the Fe_3_O_4_(4-40) diffraction peak was not observed in the ϕ-scan measurement. Therefore, we concluded that the Fe_3_O_4_ had a textured structure and the growth direction was (111).

The θ–2θ XRD diffraction pattern of Fe_3_O_4_ in W/O (green line) exhibited small peaks related to Fe_3_O_4_(311), (400), (422) and unknown peaks. In a previous study^30^, the XRD of Fe_3_O_4_ on SiO_2_ indicated that the Fe_3_O_4_ layer was polycrystalline and contained other phases.

To investigate the crystallinity of the Fe_3_O_4_ layer in detail, we carried out X-ray reciprocal space mapping around the symmetric (222) diffraction for Fe_3_O_4_ in EPI and AMO (Fig. 3(d)). The symmetrical scan showed that the Fe_3_O_4_(222) spot on amorphous-Al_2_O_3_ was larger than the Fe_3_O_4_ spot on γ-Al_2_O_3_, which means that the Fe_3_O_4_ in AMO had an angle distribution in the growth directions. Although the reason for the (111) oriented Fe_3_O_4_ growth on amorphous-Al_2_O_3_/Si(111) was unclear, two possibilities exist that could explain this growth. The first is a reduction in the total anisotropy energy related to the surface energy and interface energy^31^. The difference between AMO and W/O could be attributed to the difference of the surface and interface energy of amo-Al_2_O_3_ and amo-SiO. The second possibility is that the amo-Al_2_O_3_ maintains a crystal structure of Si locally because the amo-Al_2_O_3_ layer was very thin. Fe_3_O_4_ could utilize such a microcrystal-like region as a growth nucleus.

### Transmission electron microscope observation

**Figure 4:**
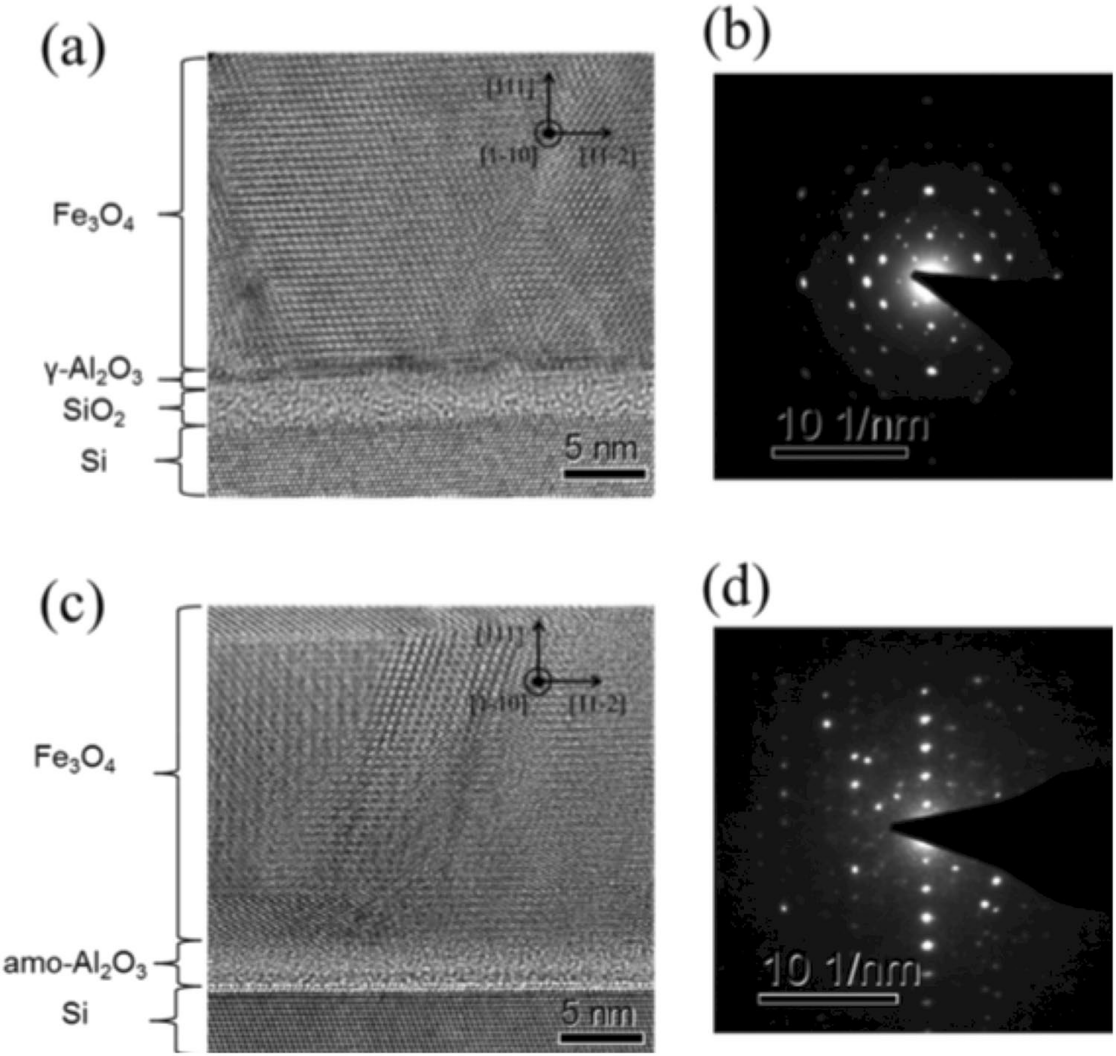
TEM observation: (**a**) Cross-section TEM image and (**b**) electron diffraction pattern of Si(111)/γ-Al_2_O_3_/Fe_3_O_4_ in EPI taken along the [1-10] axis zone, (**c**) Cross-section TEM image and (**d**) electron diffraction of Si(111)/amo-Al_2_O_3_/Fe_3_O_4_ in AMO taken along the [1-10] axis zone.

We conducted cross-sectional transition electron microscopy (TEM) analysis to confirm the crystallinity and compositions of the materials. Figure 4 shows the cross-section TEM images in which the electron beams were incident along the Si [1-10] zone axis. In Fig. 4(a), the TEM image shows that the Fe atoms of Fe_3_O_4_ were orderly aligned; thus, the Fe_3_O_4_ film was epitaxial. The electron diffraction (ED) of Fe_3_O_4_ in EPI shown in Fig. 4(b) was in good agreement with the simulation of spinel structure. The left side in Fig. 4(a) shows the epitaxial relationship on [11-2]Fe_3_O_4_(111)/[11-2]Si(111), whereas the center of image shows the epitaxial relationship on [-1-12]Fe_3_O_4_(111)/[11-2]Si(111), which were consistent with the results of the ϕ-scan measurements in the XRD. In addition, the spacing of the (111) planes were estimated at 4.87 Å from the high angle annular dark-field scanning (HAADF) image (Supplementary Fig. S4(c)), which were almost the same as the out-of-plane lattice constant (4.84 Å) determined by XRD in Fig. 3(a) and that of bulk Fe_3_O_4_ (4.85 Å). In contrast, the TEM image of Fe_3_O_4_ in AMO shown in Fig. 4(c) demonstrated that the structure was polycrystalline and grain boundaries were clearly observed. The ED image in Fig. 4(d) consisted of the diffraction from the grains with some crystal orientations. In the low magnification TEM image (supplementary Fig. S4(b)), some grains with a size of 15–30 nm appeared.

**Figure 5:**
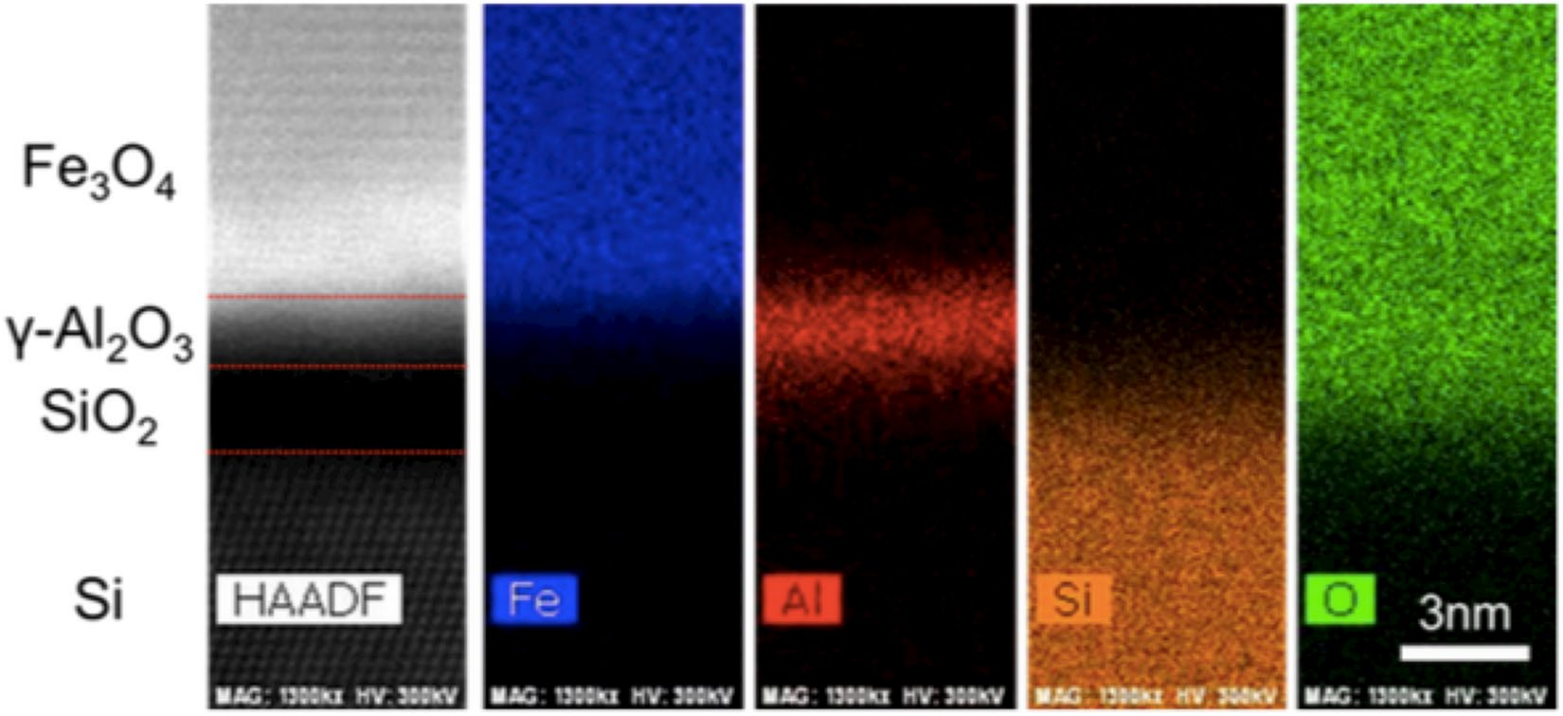
The EDS mapping of the heterostructure: From the left image, HAADF image, EDS mapping of Fe, Al, Si, and O.

With respect to the buffer layer, the thickness of γ-Al_2_O_3_ was estimated from the HRTEM image (Fig. 4(a)) to be approximately 1 nm, which was thinner than the nominal value measured by the crystal oscillator in the chamber. The reason for this difference in thickness was unclear; however, it could be due to the fluctuation of the crystal oscillator or re-evaporation of Al_2_O_3_ because the γ-Al_2_O_3_ was grown at a high temperature (900 °C). We could see the amorphous layer under the γ-Al_2_O_3_ layer, which was determined to be a SiO_x_ layer by HAADF and Energy dispersive X-ray spectroscopy (EDS) mapping images (Fig. 5). The SiO_x_ layer was considered to form during the growth of Fe_3_O_4_ because the Fe_3_O_4_ was grown in 4 × 10^−4^ Pa O_2_ gas. It was reported that Si was oxidized through the γ-Al_2_O_3_ layer of less than 2.0 nm by introducing oxygen (>10^−3^ Pa)^32^. To confirm that, we fabricated a γ-Al_2_O_3_ (7.5 nm) film on Si(111), and carried out XRD and TEM observations (supplementary Fig. S5(a) and (b)). The γ-Al_2_O_3_ grew epitaxially on Si and we found no amorphous layer at the Si(111)/γ-Al_2_O_3_(7.5 nm) interface.

### Magnetic characteristics

**Figure 6:**
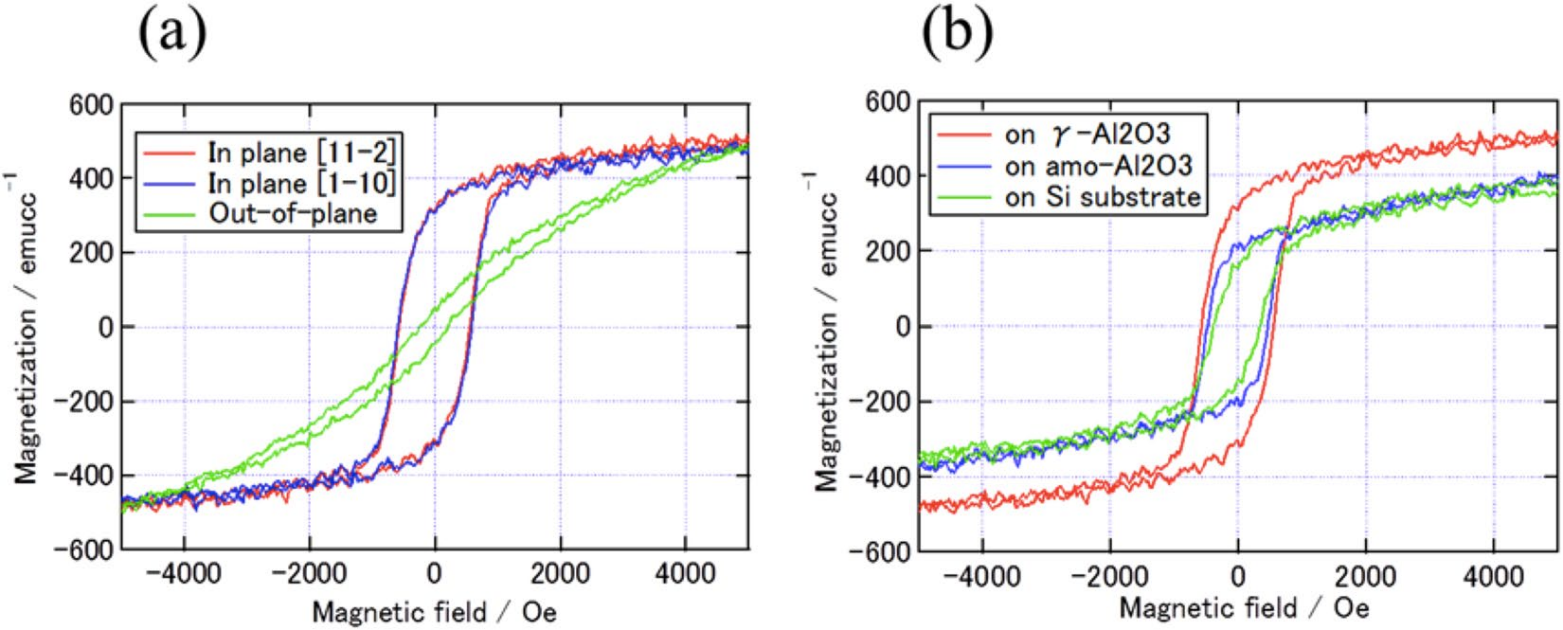
Magnetic properties (**a**) Magnetization curves of Fe_3_O_4_ on γ-Al_2_O_3_ in the magnetic field along [11-2], [1-10], and out of plane [111] (**b**) Magnetization curves of EPI, AMO, and W/O in the magnetic field along [11-2].

The magnetic character of Fe_3_O_4_ is one of its fundamental properties. The magnetization curves at room temperature for the Fe_3_O_4_ films on γ-Al_2_O_3_ layer are shown in Fig. 6(a). The directions of the magnetic field were in-plane [11-2], in-plane [1-10] and out-of-plane [111]. The hysteresis curve along [11-2] was the same as that along [1-10] and the Fe_3_O_4_ film had in-plane magnetization. The saturation magnetization (M_s_) was 480 emu/cm^3^ for all magnetic field directions. The remanent magnetization (M_r_), the coercive field (H_c_), and the remanent ratio (M_r_/M_s_) in the in-plane field were 280 emu/cm^3^ 500 Oe, and 0.48, respectively, and those for the out-of-plane field were 47 emu/cm^3^, 225 Oe, and 0.08, respectively. The hysteresis loops for Fe_3_O_4_ in EPI, AMO, and W/O are illustrated in Fig. 6(b). Fe_3_O_4_ in EPI had the largest H_c_ and M_s_ among the three samples. The M_s_ of Fe_3_O_4_ in EPI was the same as the value of bulk Fe_3_O_4_. Although the reason for small magnetization for AMO and W/O has not been clear so far, the antiphase boundary or disordered structure at grain boundary could be responsible for it^33, 34^.

### Transport characteristics

**Figure 7:**
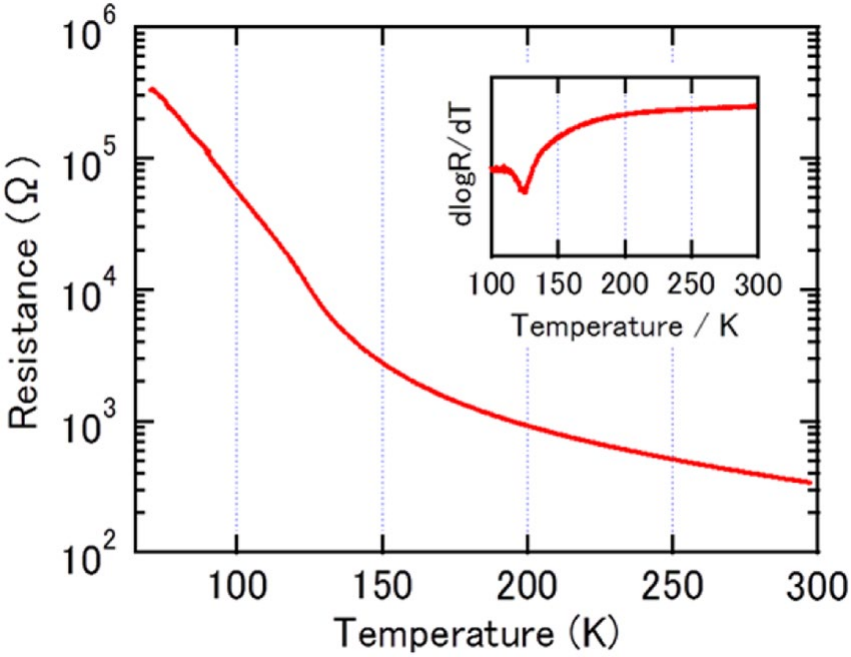
Transport properties: The resistivity of Si(111)/γ-Al_2_O_3_/Fe_3_O_4_ as a function of temperature. The inset is dlogR/dT plot.

Figure 7 shows that the dependence of the resistance on temperature for the Fe_3_O_4_ film in EPI. As is well-known, Fe_3_O_4_ is an electric conductor at room temperature and the resistivity increases exponentially with decreasing temperature. The resistivity of the film at 300 K was 2.5 × 10^−4^ Ωcm, which is lower than the bulk value (5 × 10^−3^ Ωcm)^35^. The dlogR/dT plots (inset) show a valley at approximately 120 K. This anomaly corresponds to a Verwey transition^36^, which is a famous phase transition in Fe_3_O_4_. The Verwey transition has been reported to sharply change the resistivity by approximately one digit^37^; however, the transition is easily disappeared by impurities or structure defects^34, 38^. As the Fe_3_O_4_ in EPI possessed magnetic and electric characteristics that were comparable to bulk Fe_3_O_4_, the Fe_3_O_4_ on γ-Al_2_O_3_ buffer layer was very good quality.

## Conclusions

We fabricated an epitaxial Fe_3_O_4_ film on a Si substrate by inserting an γ-Al_2_O_3_ buffer layer. From the XRD measurement and TEM observation, the γ-Al_2_O_3_ buffer layer contributed to the growth of epitaxial Fe_3_O_4_(111) on Si(111). In contrast, the Fe_3_O_4_ film on an amo-Al_2_O_3_ buffer layer had an (111)-orientation with a textured structure. The Fe_3_O_4_ on γ-Al_2_O_3_ had magnetic properties corresponding to the bulk Fe_3_O_4_, furthermore the resistivity exhibited a Verwey transition at 120 K. The results indicate that the heterostructure of Si substrate/γ-Al_2_O_3_/Fe_3_O_4_ could be used as a part of magnetic tunnel junctions or spin injection devices and will allow us to integrate spintronic devices including Fe_3_O_4_ electrode, e.g., spin-FET or magnetic tunnel junctions, on Si.

## Methods

### Preparation of the samples

Before deposition, the Si substrate was cleaned by a standard Radio Corporation of America clean^39^ and hydrofluoric (HF) acid solution and annealed at 900 °C under a vacuum of < 10^−6^ Pa[29]. The γ-Al_2_O_3_ buffer layer was formed by evaporating the Al_2_O_3_ source material at 900 °C and annealing at 900 °C for 30 minutes. In previous reports, γ-Al_2_O_3_ was grown at >850 °C and under a vacuum of <10^−6^ Pa^40^. The growth conditions we used for γ-Al_2_O_3_ were in the range of the report. In Si(111)/amo-Al_2_O_3_/Fe_3_O_4_, the amo-Al_2_O_3_ was grown at room temperature under a vacuum of <3 × 10^−6^ Pa. Then, the Fe_3_O_4_ film was formed by reactive deposition at 300 °C under a O_2_ atmosphere of 4.0 × 10^−4^ Pa^41^. All the samples were fabricated under the same growth conditions to investigate the dependence of the quality of Fe_3_O_4_ films on the buffer layer.

### Measurements

The epitaxial growth and crystal structure were confirmed by RHEED, XRD (Rigaku SmartLab (9 kW)), and TEM (FEI Titan3 G2 60-300). Cross-sectional samples for TEM were prepared by using conventional mechanical polishing and dimpling techniques^42^. The magnetic properties of Fe_3_O_4_ were measured by vibrating sample magnetometer (VSM) and the electrical properties were measured by direct current (DC) measurements.

## Electronic supplementary material

Supplementary Information
