# Supplementary material for: Fabrication of Epitaxial Fe3O4 Film on a Si(111) Substrate
Source: Sci Rep. 2017 Aug 1;7:7009. doi: 10.1038/s41598-017-07104-z (PMC5539154; doi:10.1038/s41598-017-07104-z)
Supplement: Supplementary file 1 — Supplementary Information [file 41598_2017_7104_MOESM1_ESM.pdf]

Supplementary Information

Fabrication of Epitaxial Fe_3O_4 Film on a Si(111) Substrate

Nozomi Takahashi, Teodor Huminiuc, Yuta Yamamoto, Takashi Yanase,

Toshihiro Shimada, Atsufumi Hirohata, and Taro Nagahama

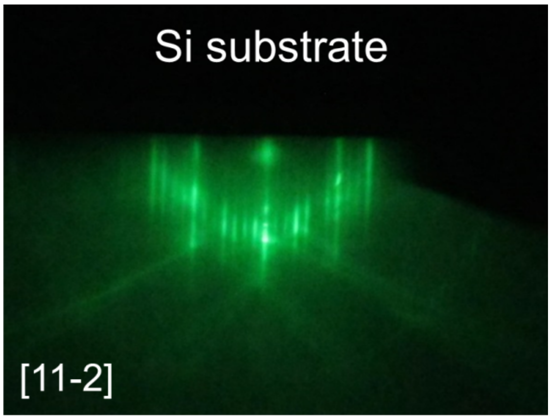

Supplementary Figure S1: RHEED pattern of the Si(111) substrate.

The electron beam was along the [11-2] direction. The RHEED pattern was taken after the treatment and annealing. The RHEED pattern shows a clear 7×7 streak, which indicates that the oxidized silicon was removed.

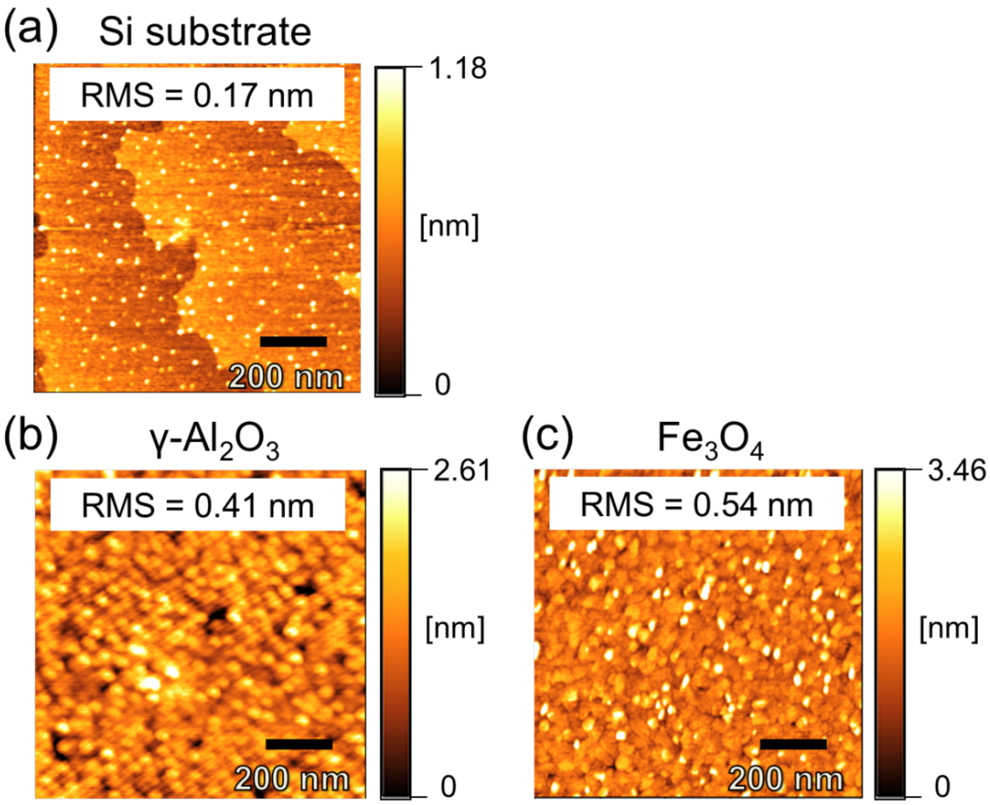

Supplementary Figure S2: AFM images of (a) Si, (b) γ-Al_2O_3 and (c) Fe_3O_4.

The roughness of root mean square (R_{rms}) value of the Si substrate was estimated to be 0.17 nm (Fig. S2 (a)). The image has a terrace-and-step structure. The R_{rms} of γ-Al_2O_3 was estimated to be 0.41 nm (Fig. S2 (b)). The surface of the γ-Al_2O_3 film consists of grains, sized 40–50 nm. The R_{rms} of Fe_3O_4 was estimated to be 0.54 nm (Fig. S2 (c)). The grain size in Fig. S2 (c) was the same as that in Fig. S2 (b).

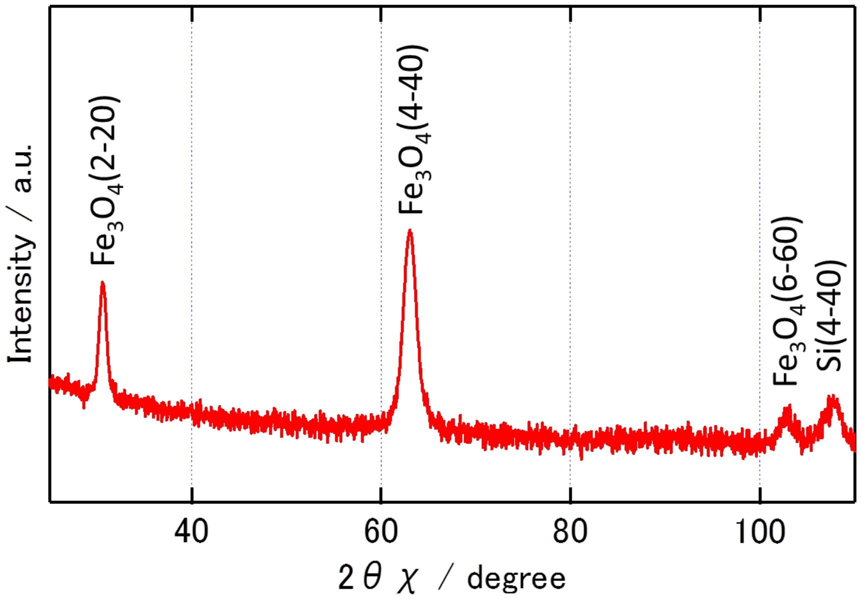

Supplementary Figure S3: In plane XRD profile of the Fe_3O_4 film.

The in-plane X-ray diffraction profile for Si(111) / γ-Al_2O_3 / Fe_3O_4 exhibited three peaks (30.6°, 63.04°, 102.76°, respectively). They were assigned as the diffraction peaks of Fe_3O_4(2-20), (4-40) and (6-60), respectively, which indicated that the Fe_3O_4 film grew epitaxially on γ-Al_2O_3.

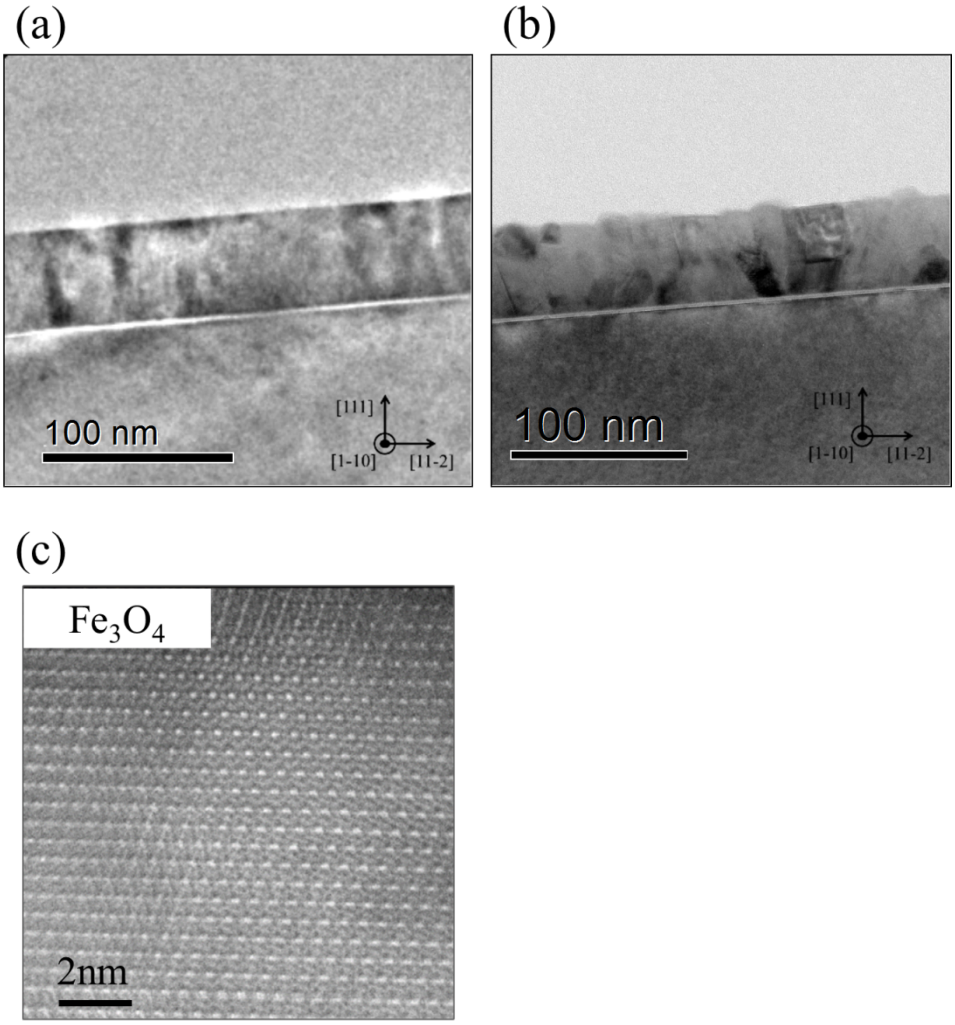

Supplementary Figure S4: TEM images of (a) Si(111) / γ-Al_2O_3 / Fe_3O_4 and (b) Si(111) / amo-Al_2O_3 / Fe_3O_4 heterostructure. (c) High resolution HAADF image of the Fe_3O_4 on γ-Al_2O_3 buffer layer.

The TEM and HAADF images were taken along the [1-10] zone axis. The Fe_3O_4 film on a γ-Al_2O_3 buffer layer in Fig. S4(a) was homogeneous, whereas grain boundaries were observed in the Fe_3O_4 film on amo-Al_2O_3, as shown in S4(b). The strong contrast in Fig.4(a) and (b) could be attributed to the thickness fluctuation of the samples.

Figure S4 (c) shows that the Fe atoms were aligned in an orderly manner. The intervals of the atoms corresponded to the Fe_3O_4 lattice parameter.

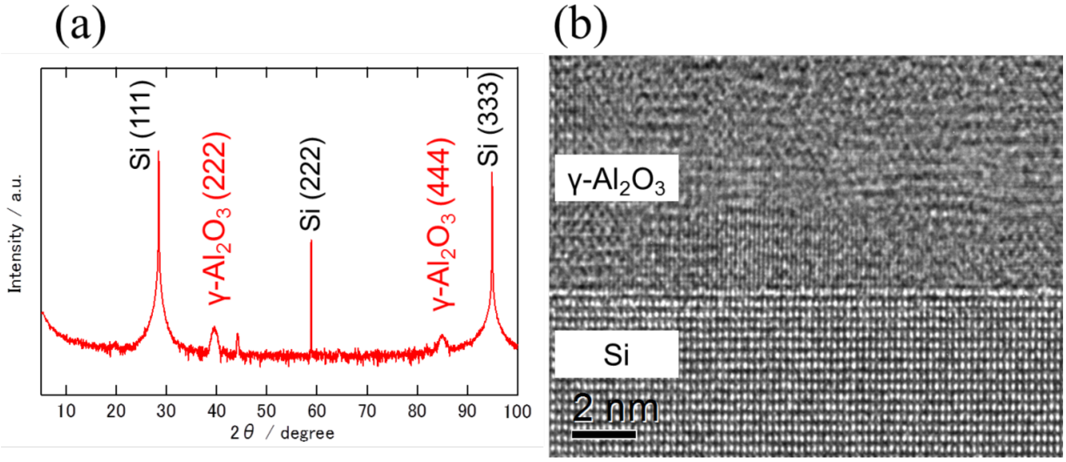

Supplementary Figure S5: (a) X-ray diffraction profile and (b) TEM image of Si(111) / γ-Al_2O_3 (7.5 nm)

Figure S5 (a) exhibited two peaks (39.58°, 84.92°) corresponding to the diffraction of γ-Al_2O_3(222) and (444), respectively, which indicated that the γ-Al_2O_3 film only had a (111) orientation and no other phases.

Figure S5 (b) was taken along the [11-2] zone axis. It showed that the γ-Al_2O_3 was a single crystal and the SiO_x did not exist at the interface of Si(111) / γ-Al_2O_3 (7.5 nm).

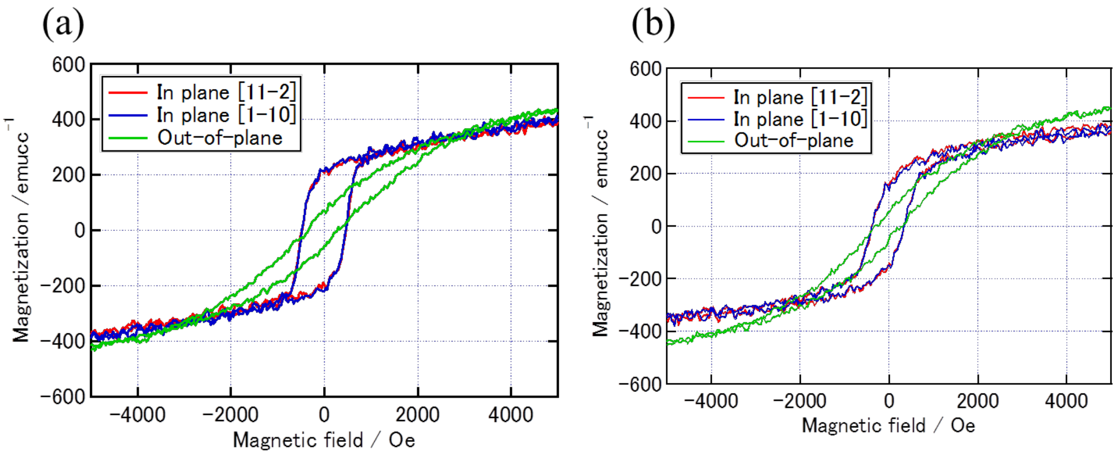

Supplementary Figure S6: Room temperature magnetization loops of (a) Fe_3O_4 on amo-Al_2O_3 and (b) Fe_3O_4 on Si substrate

The directions of the magnetic field were in-plane [11-2], in-plane [1-10] and out-of-plane [111]. Both Fe_3O_4 on the γ-Al_2O_3 buffer layer and Fe_3O_4 on the Si substrate have in-plane magnetization. The saturation magnetization (Ms) of samples (a) and (b) was approximately 400 emu/cm^3.
